# The Use of Polymer Membranes for the Recovery of Copper, Zinc and Nickel from Model Solutions and Jewellery Waste

**DOI:** 10.3390/polym15051149

**Published:** 2023-02-24

**Authors:** Elżbieta Radzymińska-Lenarcik, Ilona Pyszka, Włodzimierz Urbaniak

**Affiliations:** 1 Institute of Mathematics and Physics, Bydgoszcz University of Science and Technology, 85-796 Bydgoszcz, Poland; 2 Faculty of Chemical Technology and Engineering, Bydgoszcz University of Science and Technology, 85-326 Bydgoszcz, Poland; 3 Faculty of Chemistry, Adam Mickiewicz University, 61-614 Poznan, Poland

**Keywords:** copper(II), zinc(II), nickel(II), Cyphos IL, polymer inclusion membrane, jewellery waste

## Abstract

A polymeric inclusion membrane (PIM) consisting of matrix CTA (cellulose triacetate), ONPPE (o-nitrophenyl pentyl ether) and phosphonium salts (Cyphos 101, Cyphos 104) was used for separation of Cu(II), Zn(II) and Ni(II) ions. Optimum conditions for metal separation were determined, i.e., the optimal concentration of phosphonium salts in the membrane, as well as the optimal concentration of chloride ions in the feeding phase. On the basis of analytical determinations, the values of parameters characterizing transport were calculated. The tested membranes most effectively transported Cu(II) and Zn(II) ions. The highest recovery coefficients (RF) were found for PIMs with Cyphos IL 101. For Cu(II) and Zn(II), they are 92% and 51%, respectively. Ni(II) ions practically remain in the feed phase because they do not form anionic complexes with chloride ions. The obtained results suggest that there is a possibility of using these membranes for separation of Cu(II) over Zn(II) and Ni(II) from acidic chloride solutions. The PIM with Cyphos IL 101 can be used to recover copper and zinc from jewellery waste. The PIMs were characterized by AFM and SEM microscopy. The calculated values of the diffusion coefficient indicate that the boundary stage of the process is the diffusion of the complex salt of the metal ion with the carrier through the membrane.

## 1. Introduction

Separation of metal ions and low-molecular-weight organic compounds using polymer inclusion membranes has been proposed as an environmentally friendly separation technology [1,2,3,4,5,6,7]. Membrane separation has evolved into a more rapidly developing and diverse field. Today, there are many types of membranes with different applications. Membrane processes, the latest achievement in process engineering, can successfully replace many ineffective, conventional separation methods and in some cases complement them, improving the parameters of the output fluxes and the economics of the treatment [8,9,10,11,12,13]. PIMs have been applied in separation processes, e.g., precious metals [14,15], lanthanides [16], alkali metals [17,18,19] and heavy metals [18,20,21,22,23,24]. PIMs can also be used for the separation of copper(II) and zinc(II) from water and soil solutions [25,26].

A PIM consists of a carrier, a plasticizer and a matrix [7]. In recent years, ionic liquids, including quaternary phosphonium salts, have been proposed for use as ion carriers through PIMs [5,27,28,29]. Using ionic liquids such as, for example, Cyphos 101 and Cyphos 104, it is possible to remove iron and zinc ions from chloride waste aqueous solutions [5,27,28,29,30], cadmium, copper [31,32] and chromium(VI) ions [33,34], as well as to separate the noble metal Au(III) [35,36] and Pd(II) ions [37].

There is a huge demand for jewellery products in the modern world. Approximately 890 tonnes of gold jewellery products were purchased in the first half of 2022 [38]. There is also an increase in demand for jewellery made from metal alloys that do not contain gold. One such alloy is the so-called new silver (also known as high-nickel brass, argentan, paktong, alpacca and German silver). This alloy was invented in the 17th century in China. Today, it is known and used worldwide. It is particularly appreciated by jewellers because it perfectly imitates real silver. Unlike silver, new silver does not darken when exposed to the elements and does not cause allergic reactions. In addition to jewellery products, the alloy is widely used in a variety of industries, e.g., electronics, electrical engineering (production of resistors), telecommunication and radio technology, in the manufacture of precision apparatus, musical instruments, medical equipment, crockery and even in the manufacture of boat fittings [39,40].

The new silver, contrary to its name, does not contain pure silver in its composition. The chemical composition of this alloy can vary [41]. The composition of new silver according to ASTM B 122M is given in Table 1.

The main metals used are zinc, copper and nickel (Table 1). These metals are widely used in various industries. For example, copper is used in electrical engineering for the manufacture of cables and wires; in the energy and chemical industries for the manufacture of radiators, chemical apparatus and heat exchangers; and in the automotive industry [42], among others. Nickel is a very good catalyst for reduction processes, commonly used in the hydrogenation of fats and is also used as an additive in alloy steels, primarily stainless steel [43,44,45]. Zinc, on the other hand, has widespread technological applications in the galvanizing process to make steel corrosion-resistant, in the manufacture of batteries, roofing and is used as a catalyst in the production of India rubber [46]. Zinc is a component of many alloys, e.g., brass and tombac (with copper) as well as zamak (with aluminium) [47,48].

A major challenge facing the modern world is the management of scrap metal alloy waste. In principle, all metals sourced from scrapyards undergo pyrometallurgical recycling in smelters or foundries [49,50]. However, the hydrometallurgical process, also known as extractive metallurgy, is used more and more often in order to reduce air pollution from greenhouse gases [51]. Hydrometallurgical processes using the traditional liquid–liquid extraction method are used to produce gold, platinum, palladium, lanthanides, copper, cobalt and nickel [52,53]. Ionic liquids are becoming increasingly important in the extraction of metal ions [54]. In extraction, quaternary phosphonium salts are used to separate uranium(VI) and thorium(IV) from other rare elements [55], to separate cobalt from nickel, magnesium and calcium [56], as well as in the recovery of PGMs (platinum group metals) from spent catalytic converters [57] and Ag(I) from acidic nitrate environments [58].

The aim of this work is to compare the effectiveness of polymeric inclusion membranes based on CTA with phosphonium ionic liquids (Cyphos 101 and Cyphos 104) in terms of Cu(II) recovery from three-component Cu-Zn-Ni mixtures. These phosphonium ionic liquids have not yet been used as ion carriers in PIM for the separation of copper ions from the mixture of Cu(II)-Zn(II)-Ni(II) ions, as well as for the recovery of Cu(II) from waste jewellery (new silver waste). This work also aimed to determine the optimal conditions for Cu(II) separation using PIMs. Therefore, the effect of chloride ion concentration in the feed phase and carrier concentration in PIMs were studied.

## 2. Experimental Procedure

### 2.1. Materials

**Figure 1:**
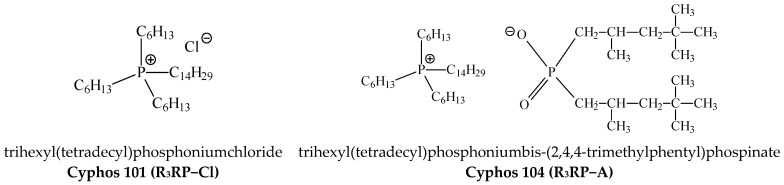
The carrier structures.

Two phosphonium ionic liquids, i.e., trihexyl(tetradecyl)phosphoniumchloride (Cyphos 101) (Figure 1) and trihexyl(tetradecyl)phosphoniumbis-(2,4,4-trimethylphentyl)phospinate (Cyphos 104) (Figure 1), cellulose triacetate (CTA), o-nitrophenylpentyl ether (ONPPE) and dichloromethane (DCM) were purchased from Sigma-Aldrich (Poznan, Poland). All inorganic reagents were purchased from POCh (Gliwice, Poland). Solutions of metal ions were prepared by dissolving appropriate amounts of metal in deionized water.

Some of ionic liquid physical properties are presented in Table 2.

**Figure 2:**
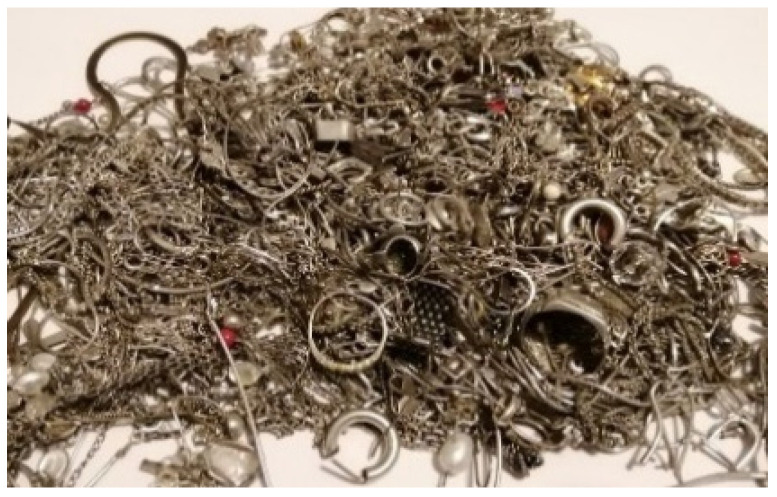
The new silver jewellery waste.

The spent jewellery waste (Figure 2) was obtained from a local market (jewellery workshops). A mass of 1g of the alloy was leached for 6 h with concentrated HCl at a temperature of 60 °C. The obtained solution was filtered and diluted to 100 cm^3^. The content of copper, zinc and nickel were determined using the AAS method. For transport tests, the solution was diluted.

### 2.2. Transport of Metal Ions across PIMs

PIMs were prepared in accordance with the previously used procedure [5,6,24,27,28,31,32]. PIMs are made of three basic substances: a polymer matrix (CTA), a carrier (phosphorus ionic liquid) and a plasticizer (ONPPE). A portion of the 3-component solution in DCM was placed in a 7.0 cm diameter petri dish. The DMC was evaporated overnight, and the resulting film was separated from the glass plate. The membranes were soaked in distilled water to achieve homogeneity. The effective membrane area exposed to the two phases was 4.9 cm^2^.

Surface characterization of PIMs was performed by atomic force microscopy (AFM) using an atomic force multimode scanning probe microscope IIIa (Digital Instruments Vecco Metrology Group, Santa Barbara, CA, USA).

In addition, PIMs were characterized using scanning electron microscopy (SEM). SEM images of the films were acquired using a Hitachi SU 3500 SEM/EDS (energy dispersive spectroscopy) microscope operated at 10.0 kV. Membranes are visualized in 5.0 × 5.0 μm^2^ images.

The thickness of the PIM was measured with a digital micrometer (Panametrics^®^ Magna-Mike^®^ 8500 (San Diego, CA, USA)) with an accuracy of 0.1 µm.

The feed phase was an aqueous chloride solution of copper(II), zinc(II) and nickel(II) ions with a concentration of C_0_ = 0.01 mol/dm^3^ for each metal ion. The feed phase is buffered at pH = 2 by adding the appropriate amount of HCl. The receiving phase was 2M HCl. The concentrations of the metal ions were measured over a specific time of transport at 324.7, 213.9 and 232.0 nm for copper, zinc and nickel, respectively.

### 2.3. Calculation Formulae

Transport through PIMs can be described by initial flux (J_0_), selectivity coefficient (S) and recovery coefficient (RF, %) [59,60,61]. Table 3 summarizes the formulae used to calculate transport efficiency as well as to characterize membranes

## 3. Results and Discussion

### 3.1. Membrane Characteristics

**Figure 3:**
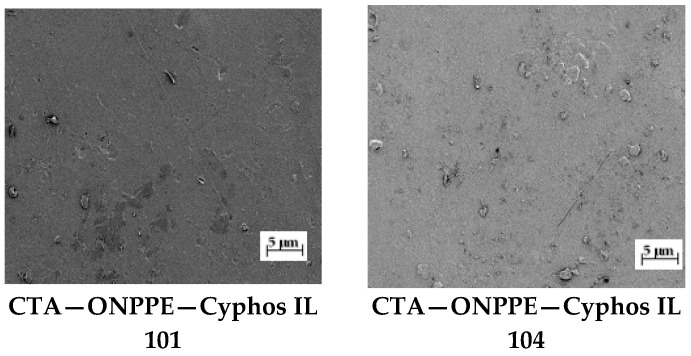
SEM picture of PIMs. Magnification ×150.

**Figure 4:**
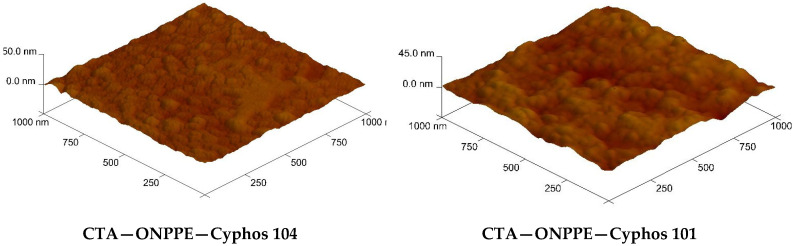
AFM images of PIMs with Cyphos IL.

The microscopic images of the CTA-ONPPE-carrier membrane are shown in Figure 3 and Figure 4.

The CTA membrane has well-defined pores that disappear completely after the addition of a plasticizer (ONPPE) and a carrier (Cyphos 101 and 104) (Figure 3). Similar images were obtained by Arous et al., who studied the structure of membranes based on CTA with tributyl phosphate (TBP) as a carrier and o-NPOE (o-nitrophenyl octyl ether) as a plasticizer [62]. The plasticizer is an integral part of the membrane. Plasticizers have the ability to interact with polymer chains, resulting in the desired flexibility and smoothness of the system [2,7,62].

The membrane had a dense and homogeneous structure. Furthermore, the roughness of the film surface could be visually confirmed in the image. Carriers were able to crystallize in the membrane. Their molecules migrated to the membrane surface, causing the roughness and porosity of the membrane. Mahanty [63] and Ugur [20] also found no pores in polymer inclusion membranes in SEM images shown at the µm scale.

The membrane roughness was calculated using NanoScope v.5.12 AFM image processing software from AFM images using the definitions shown in Table 3 (Equations (6) and (7)), and they are presented in Table 4.

As can be seen (Table 4), membranes containing Cyphos 101 as a carrier are characterized by higher roughness compared to membranes with Cyphos 104.

### 3.2. The Effect of Carrier Concentration on Cu(II) Transport through PIMs

In order to determine the effect of the type and amount of carrier on the rate of Cu(II) transfer through PIMs, transport processes were carried out using CTA membranes with Cyphos IL 101 and Cyphos IL 104 as carriers. Table 5 and Table 6 show the percentage composition of the membranes and the rate constants for the reaction of Cu(II) with both carriers during transport through PIMs.

The membrane thickness was also measured at at least 20 different points of the membrane surface and presented in Table 5 and Table 6. As the percentage of the carrier in PIMs increases, the thickness of the membranes also increases (Table 5 and Table 6). Thickness of the PIM with Cyphos 101 ranges from 25.1 μm (15% IL) to 45.7 μm (60% IL). Depending on the percentage of carrier in the membrane, the thickness of the PIM with Cyphos 104 ranges from 31.4 to 52.1 μm. The greater thickness of the PIM with Cyphos 104 can be explained by the higher molar mass of this carrier.

For both carriers, the highest values of reaction rate constant (k) were recorded for membranes with a composition of 55 wt.% carrier content, 40 wt.% CTA and 5 wt.% plasticizer (Table 5 and Table 6). Further research was conducted using membranes with the above composition.

### 3.3. Effect of Cl^−^ Ion Concentration in the Feed Phase

**Figure 5:**
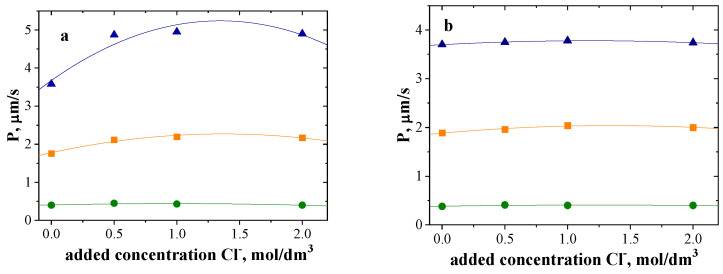
The dependence on the permeability coefficients as the function of Cl^−^ concentration; the feed phase: ▲Cu(II), ■Zn(II) and ●Ni(II) in HCl; the receiving phase: 2M HCl. The initial concentration of each metal ion was 0.01 M. (**a**) PIM with Cyphos 101. (**b**) PIM with Cyphos 104.

Next, the influence of chloride ion concentration in the feed phase on the transport of Cu(II), Zn(II) and Ni(II) was studied. The concentration of chloride ions was increased in the feed phase by adding an appropriate amount of 4M NaCl solution. Figure 5 shows the dependence on the permeability coefficients for metal ion transport (P, μm/s) as the function of Cl^−^ concentration.

In the case of the membrane with Cyphos 101, slightly increasing the concentration of added chloride ions in the feed phase (from 0 to 0.5 mol/dm^3^) increases the permeability coefficient, especially of the Cu(II) and Zn(II) ions (Figure 5). Further increases in Cl^−^ ion concentration do not result in significant differences in metal ion transport (Figure 5a). In the case of the PIM with Cyphos 104, increasing the added concentration of Cl^−^ ions in the feed phase does not significantly affect the transport of each metal ion (Figure 5b). For both tested membranes, Ni(II) was transported in a negligible amount.

In further tests, the concentration of added chloride ions in the feed phase was 0.5 mol/dm^3^.

### 3.4. Separation of Copper(II), Zinc(II) and Nickel(II) Ions Using PIMs with Cyphos IL

Competitive transport of investigated metal ions from aqueous chloride solutions through CTA polymer membrane with Cyphos IL into 2 mol/dm^3^ HCl was investigated.

**Figure 6:**
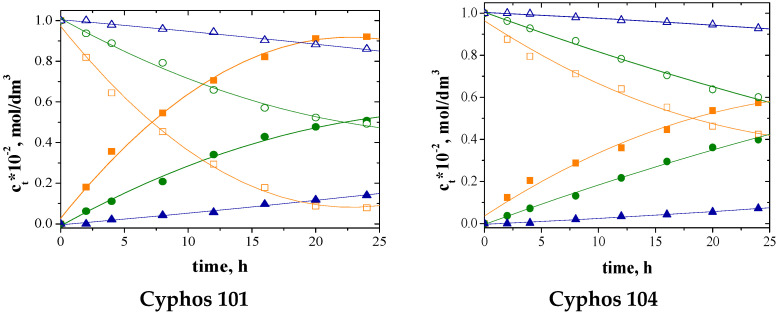
The concentration of metal ions in the feed and receiving phases during transport across PIM with Cyphos; feed phase: ☐—Cu, ○—Zn, △—Ni; receiving phase: ■—Cu(II), ●—Zn(II), ▲—Ni(II).

The change in the concentration of metal ions, both in the feed and receiving phase over time, is shown in Figure 6.

Parameters characterizing transport calculated on the basis of analytical determinations from Equations (3) and (4) (Table 3) are presented in Table 7.

The data in Table 7 show that the initial Cu(II) flux values are the highest for all supports, indicating that Cu(II) ions are transported at the highest rate. Ni(II) ion transport is the least. The initial value of the metal ion transport flux also depends on the type of carrier used. The Cyphos 101 PIM showed the highest initial flux values. In the case of PIM with Cyphos 104, the initial fluxes of Zn(II) and Cu(II) ions are slightly lower than for the membrane with Cyphos 101. However, for Ni(II) ions, these values are comparable. It can also be speculated that the higher volume of anions (A) in Cyphos IL 104, and thus their lower mobility, lowers J_0_ values.

Comparing the values of the initial fluxes (Table 7) with the roughness values (Table 4) of the membranes, it can be concluded that the increase in roughness increases the transport of each metal ion, which is consistent with the literature data [64]. According to the literature, membranes with a more heterogeneous surface show a greater capacity to transport metal ions compared to homogeneous membranes [64,65].

According to Arous [62,65], the amorphous structure of a PIM indicates that the compound is transported through the membrane by diffusion of the metal–carrier complex. At the interface of the feeding phase and the PIM surface, metal ions combine with carrier molecules present in the membrane to form complex salts. The complex salt diffuses to the opposite surface of the membrane, where metal ions are released to the receiving phase. The reaction responsible for ion transport involves anion exchange. This exchange is more efficient when CYPHOS 101 (Table 7) is used as a carrier, perhaps due to the more mobile chloride anion.

**Figure 7:**
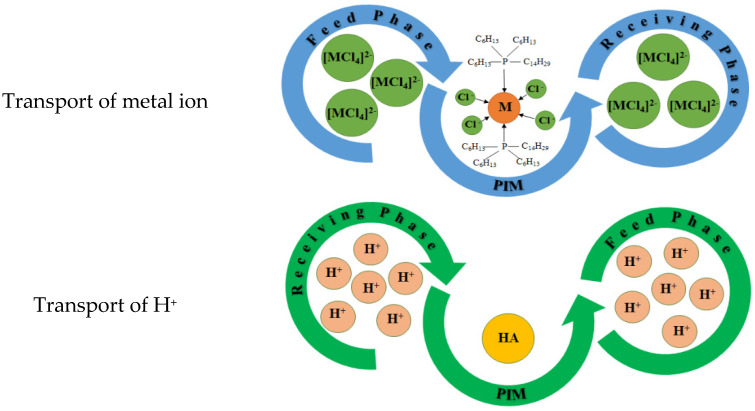
Schematic transport across PIMs with Cyphos IL.

Figure 7 presents the proposed mechanism of the transport of metal ions through PIMs with Cyphos IL.

The proposed transport mechanism can be described as follows:

At the interface feed phase/membrane, the Cu(II) and Zn(II) ions with carrier molecules form complex salts (Equation (9)):

For Cyphos IL 101: [MCl_4_]^2−^ + 2 R_3_RP-Cl ↔ (R_3_RP)_2_MCl_4_ + 2 Cl^−^
 For Cyphos IL 104: [MCl_4_]^2−^ + 2 R_3_RP-A ↔ (R_3_RP)_2_MCl_4_ + 2 A^−^ (9)

At the interface membrane/receiving phase:

For Cyphos IL 101: (R_3_RP)_2_MCl_4_ ↔ [MCl_4_]^2−^ + 2 R_3_RP-Cl   For Cyphos IL 104: (R_3_RP)_2_MCl_4_ ↔ [MCl_4_]^2−^ + 2 R_3_RP-A (10)

At the same time, hydrogen ions combine with the anions of the studied ionic liquids (A) and are transported from the receiving phase to the feed phase.

Cu(II) and Zn(II) ions are effectively transported to the receiving phase (Equations (9) and (10)), as they can form [MCl_4_]^2–^ chloro-complexes in the feed phase. Ni(II) ions are present in chloride solutions as Ni^2+^ and NiCl^+^ [66] and are incapable of forming anionic complexes. Regardless of the type of carrier used, transport of Ni(II) through the PIM with both carriers is inefficient, as evidenced by the low J_0_ values (Table 7).

### 3.5. Metal Ion Diffusion Coefficients

**Figure 8:**
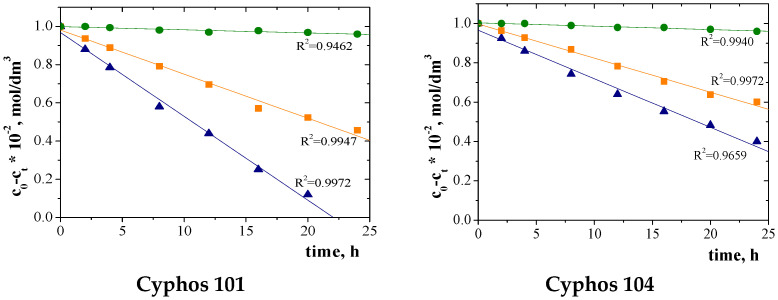
Plots of (C_0_ − C_t_) against time for ▲Cu(II), ■Zn(II) and ●Ni(II) ion transport across PIMs with Cyphos IL.

Figure 8 shows plots of (C_0_ − C_t_) as a function of time for the transport of each metal ion through PIM with Cyphos IL. These graphs were used to calculate the ion diffusion coefficients from Equation (8) (Table 3).

The values of diffusion coefficients are presented in Table 8.

The values of diffusion coefficients range from 10^−7^ to 10^−8^ cm^2^/s (Table 8) and are comparable to the diffusion coefficients of metal ions through the membrane CTA presented in the literature [59,67,68].

### 3.6. Recovery of Metal

**Figure 9:**
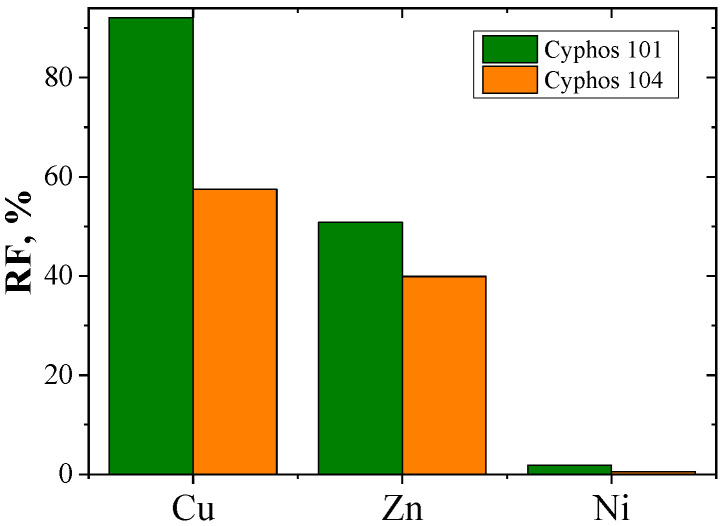
Recovery coefficient (RF) of copper, zinc and nickel after 24-hour transport through PIMs with Cyphos IL.

The recovery coefficient (RF) was calculated from Equation (5) (Table 3). Figure 9 shows the RF coefficients of metal ions.

The values of the recovery coefficient (RF) depend on the carrier used in the membrane (Figure 9). The highest recovery coefficients (RF) were found for PIMs with Cyphos IL 101. For Cu(II) and Zn(II), they are 92% and 51%, respectively. For PIMs with Cyphos IL 104, the RF values of Cu(II) and Zn(II) ions are 58% and 40%, respectively. The RF value for Ni(II) ions did not exceed 2% (PIMs with Cyphos IL 101). These ions practically remain in the feed phase.

### 3.7. Recovery of Metal from Jewellery Waste Solutions

In the next step, attempts have been made to utilize the PIMs with Cyphos IL 101 for the separation of the Cu(II) and Zn(II) ions from jewellery waste. The content of Cu(II), Zn(II) and Ni(II) in the tested sample of new silver waste was 56%, 20% and 7.5%, respectively.

The copper(II), zinc(II) and nickel(II) concentrations in the starting solution for transport tests were 0.008 mol/dm^3^, 0.003 mol/dm^3^ and 0.001 mol/dm^3^, respectively. The values of initial fluxes (J_0_) for the transport of metal ions from the solution after leaching waste new silver, the selectivity coefficient (S) and the recovery coefficient (RF) are shown in Table 9.

Compared to the model solution, in which the concentrations of all metal ions were equal (Table 6), in the real solution, which is dominated by copper, J_0_ for Cu(II) has a higher value, while J_0_ for Zn(II) has a slightly lower value (Table 9). The initial flux of Ni(II) ions is negligible. This increases the Cu(II)/Zn(II) separation coefficient to almost 2.5 and enables selective separation of Ni(II) ions from Cu(II) and Zn(II) ions. Ni(II) ions remain in the feed phase.

The recovery coefficient values of Cu(II) and Zn(II) ions are 90% and 35%, respectively.

## 4. Conclusions

The polymer inclusion membranes with Cyphos IL 101 and Cyphos IL 104 can be used due to their properties for the separation of copper(II), zinc(II) and nickel(II) ions. For all carriers, the initial fluxes of Cu(II) and Zn(II) have the highest value.

The transport efficiency of Cu(II) and Zn(II) ions facilitates the formation of [MCl_4_]^2−^ chloro-complexes in the chloride feeding phase.

Diffusion coefficient values show that the limiting step of the process is the transfer of the metal complex through the membrane barrier.

The highest recovery coefficients (RF) were found for PIMs with Cyphos IL 101. For Cu(II) and Zn(II), they are 92% and 51%, respectively. The RF value for Ni(II) ions did not exceed 2% (PIMs with Cyphos IL 101). These ions practically remain in the feed phase because they do not form anionic complexes with chloride ions.

The obtained results suggest that there is a possibility of application of these membranes for separation of Cu(II) over Zn(II) and Ni(II) from acidic chloride solutions.

The PIM with Cyphos 101 can be used to recover copper and zinc from jewellery waste. Copper(II) can be selectively transferred from the feed containing high Cu(II) concentration and the low concentration of Zn(II) ions. In this case, the recovery coefficient values of Cu(II) and Zn(II) ions are 90% and 35%, respectively.

From a practical point of view, the Cyphos IL 101 PIM can be used for the selective separation of Cu(II), Zn(II) and Ni(II) ions from waste containing these metals.

## Figures and Tables

**Table 1:** Chemical composition of new silver [41].

Name	Cu	Ni	Zn	Pb	Fe	Mn	Sn	Other
Concentration [%]	63–66	11–13	20–25	0.03	0.3	0.5	0.03	0.2

**Table 2:** Physical properties of phosphonium ionic liquid used as carriers in tested PIMs.

Physical Properties	CYPHOS 101	CYPHOS 104
Formula	C_32_H_68_ClP	C_48_H_102_O_2_P
Representation	R_3_RP−Cl	R_3_RP−A
Molecular mass g/mol	519.31	773.27
Density at 20 °C, g/cm^3^	0.882	0.892
Viscosity at 25 °C, cP	1824	707
Colour and form	Colourless liquid	Dark brown liquid

**Table 3:** The formulae used to calculate transport efficiency as well as to characterize membranes.

No.	Formula	
(1)	$\ln\left(\frac{c_{t}}{c_{0}}\right)=-kt$	*c*_0_—initial metal ion concentration (M); *c_t_*—metal ion concentration at a given time in the feed phase (M); *k* is the rate constant (s^−1^), which could be evaluated by plotting (C_0_ − C_t_) against time; *t*—time of transport (s)
(2)	$P=-\frac{V}{A}\;\cdot k$	*P*—permeability coefficient; *V*—volume of the aqueous feed phase (m^3^); *A*—effective area of the membrane (m^2^)
(3)	$J_{0}=P\cdot c_{0}$	*J*_0_—initial flux (mol/sm^2^)
(4)	$S=\frac{J_{0,\;M_{1}}}{J_{0,\;M_{2}}}$	*S*—selectivity coefficient
(5)	$RF=\frac{c_{0}-c}{c_{0}}\;\cdot 100\%$	*RF*—recovery coefficient
(6)	$R_{a}=\frac{1}{N}\sum_{j=1}^{N}\left|Z_{j}\right|$	*R_a_*—surface average roughness; *Z_j_*—the current surface height value; *N*—number of points measured *R_q_*—root mean square roughness
(7)	$R_{q}=\sqrt{\frac{\sum_{j=1}^{N}\left|Z_{j}\right|}{N}}$	
(8)	$D_{0}=\frac{d}{\Delta_{0}}$	*D*_0_—diffusion coefficients of metal ions; *d*—thickness of membrane; Δ_0_ could be evaluated by plotting *(C*_0_ *− C_t_)* against time

**Table 4:** The roughness parameters for PIM with Cyphos.

Polymer Inclusion Membranes with Cyphos		
Carrier	Cyphos 101	Cyphos 104
Average roughness (*R_a_*, nm)	3.32 ± 0.02	2.67 ± 0.02
Mean square roughness (*R_q_*, nm)	4.48 ± 0.04	2.54 ± 0.03

**Table 5:** Percentage composition of the membranes, membrane thickness (d) and reaction rate constants (k) of Cu(II) ion transport through the CTA—Cyphos 101—ONPPE membrane.

CTA, wt. %	Cyphos 101, wt. %	ONPPE, wt. %	K × 10^3^, s^−1^	d, μm
100	-	-	0.42	22.5 ± 0.5
80	15	5	0.76	25.1 ± 0.6
60	35	5	1.32	33.2 ± 0.4
40	55	5	7.23	38.1 ± 0.5
35	60	5	5.06	45.7 ± 0.04

**Table 6:** Percentage composition of the membranes, membrane thickness (d) and reaction rate constants (k) of Cu(II) ion transport through the CTA—Cyphos 104—ONPPE membrane.

CTA, wt. %	Cyphos 104, wt. %	ONPPE, wt. %	K × 10^3^, s^−1^	d, μm
80	15	5	0.55	31.4 ± 0.8
60	35	5	1.06	39.3 ± 0.5
40	55	5	5.87	44.7 ± 0.8
35	60	5	2.38	52.1 ± 0.05

**Table 7:** Initial fluxes, selectivity order and selectivity coefficient for competitive transport of Cu(II), Zn(II) and Ni(II) ions across PIMs. Membrane: 55% carrier (Cyphos IL 101, Cyphos IL 104), 40% CTA, 5% ONPPE.

Carrier	Metal Ion(II)	Initial Flux J_0_ × 10^6^, mol/s·m^2^	S_Cu(II)/M(II)_
Cyphos IL101	Cu(II)	4.87	Cu(II) > Zn(II) > Ni(II) 2.3 10.8
	Zn(II)	2.12	
	Ni(II)	0.45	
Cyphos IL104	Cu(II)	3.75	Cu(II) > Zn(II) > Ni(II) 1.9 9.1
	Zn(II)	1.96	
	Ni(II)	0.41	

**Table 8:** Diffusion coefficients for transport of Cu(II), Zn(II) and Ni(II) ions through PIMs with Cyphos IL.

Carrier	Metal Ion(II)	Δ_0_, s/cm	D_0_, cm^2^/s
Cyphos IL 101	Cu(II)	228.14	1.67 × 10^−7^
	Zn(II)	43.54	8.75 × 10^−7^
	Ni(II)	-	-
Cyphos IL 104	Cu(II)	429.81	1.04 × 10^−7^
	Zn(II)	86.96	5.14 × 10^−8^
	Ni(II)	-	-

**Table 9:** Initial fluxes (J_0_), selectivity coefficients (S) and recovery coefficients (RF) of Cu(II), Zn(II) and Ni(II) ions after 24 h transport across PIMs with Cyphos IL 101; feed phase: the leaching solution (pH = 2, [Cl^−^] = 1 mol/dm^3^); receiving phase: 2M HCl.

Metal Ions	Initial Flux, J_0_ × 10^6^, mol/s·m^2^	S_Cu(II)/M(II)_	RF, %
Cu(II)	5.53	Cu(II) > Zn(II) > Ni(II) 2.46 553	90
Zn(II)	1.84		35
Ni(II)	0.01		>0.1

## Data Availability

Not applicable.
